# Cancer Care in Post-Roe America: How Do We Move Forward?

**DOI:** 10.1007/s13187-024-02512-y

**Published:** 2024-10-08

**Authors:** Emily Ramonna Smith, Georgia Robins Sadler

**Affiliations:** 1 University of California San Diego, La Jolla, CA USA; 2 UC San Diego Moores Cancer Center, La Jolla, CA USA; 3 Department of Surgery, UC San Diego School of Medicine, La Jolla, CA USA

## Abstract

In 2022, the Supreme Court overturned Roe v. Wade in the case of Dobbs v. Jackson Women's Health Organization. This ruling ended all federal protections for abortion, consequently reshaping the American healthcare landscape. Two years later, the impacts of this ruling on cancer care remain largely undiscussed. This reflection summarizes a literature review exploring the effects of the Dobbs decision on cancer treatment access in America. Articles were identified using databases such as PubMed, CINAHL, Westlaw Campus Research, Nexis Uni, Google Scholar, and JSTOR. Search terms included cancer, drugs, Methotrexate, access, treatment delays, Roe v. Wade, Dobbs, post-Roe, abortion, abortion-inducing, maternal health, reproductive rights, and limitations. Thirty eligible articles, published in English from 2001 to 2024, were reviewed in full text. The findings of this reflection article highlight the urgent need for the oncology community to understand the potential impact of these changes and work collectively to ensure the equal access to women's cancer care.

## A Decision that Rocked the World

On June 24, 2022, the United States Supreme Court ruled in the case of *Dobbs v. Jackson Women’s Health Organization*, ending all federal protections for abortion. This historic decision overturned fifty years of legal precedent established by Roe v. Wade, revoking the constitutional right to abortion and leaving the regulation of abortion access to individual states. As of August 2024, 41 out of 50 states have restrictions on abortion access [1].

In the summer of 2023, I (Smith) participated in the *National Cancer Institute-funded (R25CA221779) Multidisciplinary Educational Approach to Reduce Cancer Disparities* program at the UC San Diego Moores Cancer Center. A year after the overturn of Roe v. Wade, I was keen to study how the Dobbs decision might impact cancer care. With the opportunity to explore a research question of choice, I dove into the existing literature, discovering the troubling implications of this judicial action on multiple fronts. In the realm of cancer care, 1 in 1000 women are pregnant at the time of their cancer diagnosis. The most recent report states that 5,507,000 women were pregnant in 2019, projecting a total potential of the Dobbs decision to impact 5507 women annually [2]. Pregnancies during this time add a layer of complexity, from the point of the diagnostic process with imaging and chemicals to the delivery of optimal treatment plans. The American Cancer Society’s website summarizes that hormone therapy, chemotherapy, and high doses of radiation, generally used for cancer diagnosis and treatment, are not usually recommended during pregnancy. There is the risk that they can harm the fetus during the pregnancy and can lead to miscarriage, birth defects, slow fetal growth, or a higher risk of childhood cancer [3].

In addition, the Dobbs decision has the potential to affect more than just pregnant cancer patients. It can transform the cancer care options for all women of childbearing age. The Dobbs decision will also impact how men and women evaluate their fertility preservation options following the Alabama Supreme Court’s ruling on preserving frozen embryos (*Idaho v. United States* and *Alliance for Hippocratic Medicine v. Food and Drug Administration)* [4]. With cancer treatment options potentially becoming limited, delayed, or refused, the debate on the Dobbs decision needs to include a discussion of the potential impacts on cancer care. This essay underscores the need to better navigate the intersection of reproductive rights and cancer care within the changing political landscape.

## Will Women Still Be Able to Access Optimal Cancer Care?

In states where abortion for medical exceptions is allowed, the language of the law is often vague and untested. For example, Arizona, Florida, Wyoming, and Indiana permit abortions if the patient has “a serious risk of substantial and irreversible impairment of a major bodily function” [5]. This language lacks specific clinical definitions, leading to confusion about what conditions qualify for the exception.

Nowhere is this legislative vagueness a more significant concern than in cancer care. For instance, Methotrexate, commonly used to treat cancer, is linked to a high risk of miscarriage. Of all reproductive-aged women with a Methotrexate prescription, 18% of those prescriptions were for cancer therapy [6]. Data from *FORWARD*, *The National Databank for Rheumatic Diseases*, highlighted reports of women experiencing delays in the refilling of their Methotrexate prescriptions for diverse medical purposes following the overturn of Roe v. Wade [7]. Such delays can have life-limiting and quality-of-life consequences for women of childbearing age. In addition, some women faced distressing questioning in the course of filling their prescriptions [7]. Trust is crucial in delivering quality care; however, excessive questioning, prescription delays, and mandatory pregnancy tests may undermine the current health benefits of prompt, trusted, and uninterrupted healthcare.

Another concern in the post-Roe era is whether equitable access to cancer care can be delivered equally across all states. In states where abortion is not permitted, will women face financial and social barriers to accessing optimal medically necessary cancer care? In a case where essential cancer care has a high risk of miscarriage, teratogenic consequences for the fetus, or a medically necessary abortion, women may lack the financial means to travel out of state. These challenges are increasingly reflected in reality as the proportion of patients traveling to other states to obtain abortion care reached nearly one in five in the first half of 2023 [8]. Little is said about how the nation can minimize such health disparities while respecting each state’s judicial power as protected by the Tenth Amendment.

## Challenges and Choices

As confusion arises in the post-Roe era, healthcare professionals need to understand the clinical impacts of this ruling. Today’s climate reveals a significant gap in knowledge about how healthcare could be impacted in post-Roe America. This will be a time to monitor access to cancer care and the quality of that care. Twelve states are projected to put constitutional amendments regarding abortion access on their November 2024 ballots, indicating that the healthcare landscape is still ever-changing [9]. Now is the time to have productive conversations to navigate better the decisions that will be made later this year.

Numerous organizations concerned with medical ethics have created thoughtful, reflective responses to the Dobbs decision. The Hastings Center, a leader in the biomedical ethics community, offers multiple analytic post-Dobbs commentaries on its website, voicing their concerns about how drastic a change the healthcare field faces.

The Abortion Defense Network has developed a “Know Your State’s Abortion Laws” guide to help medical staff understand state-specific regulations. Their focus is helping clinicians understand what conduct is still permitted in their state so they can optimally advocate for themselves and their patients [10].

Hospitals and medical schools across the U.S. are implementing various strategies to adapt medical education and care delivery to the post-Dobbs era of women’s healthcare. Organizations, such as the American Society of Clinical Oncology (ASCO), have also published suggested ethical guidelines to aid practitioners in making these difficult decisions when medical ethics, legal restrictions, and personal beliefs collide [11].

Professional organizations, like the American Medical Association (AMA), advocate for consistent, evidence-based care, especially when protecting medical education. Currently, the Accreditation Council for Graduate Medical Education (ACGME) requires access to abortion training for all ob-gyn residents. However, nearly 45% of those accredited residency programs are in states that have or are likely to have abortion restrictions. Under new AMA policies, the AMA will work to ensure that all medical students and residents retain access to medication and procedural abortion training by providing support pathways for students and residents to receive training outside their home institutions [12].

These organizations demonstrate a foundational consensus that the field must brace for the drastic consequences of the Dobbs decision, and advocacy within these groups firmly pushes for reconsidering the Dobbs decision. However, they do so without a united front or a “one voice message.” With the interpretation of state law being so vague and highly case-dependent, there is no strategy for moving forward in a united approach. Yet, as the Dobbs decision increasingly becomes integrated into today’s healthcare landscape, it is time for the organizations to create a united interpretation of how the post-Dobbs restrictions will impact the healthcare of women of childbearing age and those seeking fertility preservation choices.

As healthcare providers and women across America grapple with the far-reaching potential of the Dobbs decision to impact cancer care, providers and patients coping with other health issues are likely to encounter similar impacts. For over fifty years, women lived their reproductive years under Roe v. Wade. Today, women from the pre-Roe era, the Roe era, and the evolving post-Roe era coexist, striving to understand the consequences of this shift. By actively engaging and educating themselves and the public about the latest interpretation of the law, clinicians, patients, patient navigators, scientists, and health policy advocates can work together to safeguard access to cancer care and other critical medical care. Ensuring access to optimal oncology therapies for all women is not just important; it can be a matter of life and death.
